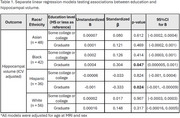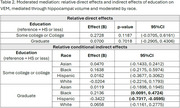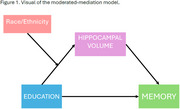# The differential association of education with hippocampal volume and episodic memory in ethnoracially diverse adults aged 90 and older

**DOI:** 10.1002/alz70857_105097

**Published:** 2025-12-25

**Authors:** Batool M. Rizvi, Alexander Ivan B. Posis, Yi Lor, Nancy X Chen, Molly R. LaPoint, Adam Brickman, María M. M. Corrada, Paola Gilsanz, Rachel A. Whitmer

**Affiliations:** ^1^ University of California, Davis, Davis, CA, USA; ^2^ Kaiser Permanente Northern California Division of Research, Pleasanton, CA, USA; ^3^ Columbia University, New York, NY, USA; ^4^ University of California, Irvine, Irvine, CA, USA

## Abstract

**Background:**

The differential association of education with brain imaging measures in late‐life is poorly characterized, particularly among diverse populations aged 90+. We examined whether education is associated with hippocampal volume and tested whether the association between education and verbal episodic memory (VEM) is mediated by hippocampal volume across race and ethnicity.

**Methods:**

We included participants from *LifeAfter90*, a diverse cohort aged 90 years and older. Education was categorized into three levels of attainment: “high school or less” (reference), “some college or college”, and “graduate”. We measured VEM with the Spanish and English Neuropsychological Scales. A 3T MRI was acquired to measure total hippocampal volumes. Linear regressions stratified by race and ethnicity tested associations of education with hippocampal volume. All regression models adjusted for age at MRI and sex/gender. We then tested whether hippocampal volume mediated the relationship between education and VEM across race and ethnicity. For the mediation analysis, we adjusted for age and MRI and the time interval between MRI and cognitive testing acquired.

**Results:**

Participants (*N* = 196) had a mean±SD age of 93.19±2.05 years, 53.6% were women, and the self‐reported race and ethnicity were 24% Asian, 21% Black, 18% Hispanic, and 33% White. Higher education (graduate) was associated with higher hippocampal volume in Black participants, but was related to lower hippocampal volume in Hispanic participants (Table 1). Education was not associated with hippocampal volume in White or Asian older adults. Hippocampal volume mediated the association between higher education (graduate) and memory performance in Hispanic and Black participants (Table 2; Figure 1). In Black participants, higher education (graduate) was associated with better episodic memory performance via larger hippocampal volume. Meanwhile, in Hispanic participants, the association of higher education (graduate) with better memory performance was suppressed (inconsistent mediation) or weakened through smaller hippocampal volume.

**Conclusions:**

Education is associated with outcomes of brain health in late‐life adulthood, particularly among Black older adults. Future work can examine whether Hispanic older adults face greater challenges during educational attainment and/or subsequent opportunities, affecting brain health outcomes in late‐life adulthood. Alternatively, Hispanic individuals may rely on cognitive reserve to maintain memory despite lower hippocampal volume.